# Quality of life assessment before and after surgery for lower limb varicose veins

**DOI:** 10.1590/1677-5449.190108

**Published:** 2020-05-08

**Authors:** Fernanda Appolonio Rocha, Esdras Marques Lins, Catarina Coelho de Almeida, Ronaldo Campelo Dias, Pedro Alberto Livino da Silva, Claudia Almeida Gameleira, Mary Gleyce de Melo Gomes Falcão, José Wellington dos Santos Barros

**Affiliations:** 1 Universidade Federal de Pernambuco – UFPE, Disciplina de Cirurgia Vascular, Recife, PE, Brasil.; 2 Universidade Federal de Pernambuco – UFPE, Hospital das Clínicas, Serviço de Cirurgia Vascular, Recife, PE, Brasil.; 3 Faculdade Pernambucana de Saúde – FPS, Curso de Medicina, Recife, PE, Brasil.; 4 Instituto de Medicina Integral Professor Fernando Figueira – IMIP, Serviço de Cirurgia Vascular, Recife, PE, Brasil.

**Keywords:** quality of life, varicose veins, venous insufficiency

## Abstract

**Background:**

Lower limb varicose veins are one of the most prevalent diseases in the global population. The disease is chronic and has a great impact on patients’ quality of life, limiting daily activities and functional performance. Several authors have emphasized the importance of including quality of life assessment in management of patients with chronic venous disease.

**Objectives:**

To evaluate quality of life before and after surgical treatment of patients with varicose veins.

**Methods:**

A before and after study design was employed. Ninety-two people with varicose veins of the lower limbs were treated surgically. Patients were divided into subsets according to age and CEAP clinical classification. Quality of life was assessed using the VEINES QOL/SYM questionnaire, administered during the preoperative period and 60 days after the operation.

**Results:**

The sample comprised 92 subjects, 82.6% (76) of whom were women and mean age was 45.7±12.11 years. CEAP class 2 was the most frequent clinical classification, in 57.6% of patients. There was a significant difference in scores before and after surgery among patients aged from 30 to 40 years. There was no difference between preoperative and postoperative scores between different CEAP groups.

**Conclusions:**

No difference in quality of life was observed after surgery in most of the patients in the present study.

## INTRODUCTION

Chronic venous disease (CVD) of the lower limbs (LL) is extremely common and has variable presentations. It is characterized by venous system dysfunction secondary to venous hypertension caused by valve incompetence and/or obstruction of venous flow. In addition to esthetic compromise, CVD can cause symptoms that result in complications and sequelae, which can have a negative influence on patients’ quality of life.^1^

The incidence of CVD increases considerably from the third decade of life onwards. In Brazil, an epidemiological study conducted by Maffei^2^ found a 35.5% prevalence of varicose veins and severe forms of CVD. This rate increases with age: the disease affects 3% of men and 20% of women in the 30-40 years age group, while at 70 years of age, 70% of the population have some degree of venous disease.^2^^,^^3^

Although surgical treatment is a widely used therapeutic option for CVD, there are few studies evaluating its impact on the quality of life (QoL) of patients who undergo surgery. There are reports of QOL assessment after surgical treatment, but in relation to other factors, such as venous hemodynamics,^4^ use of preoperative ultrasonography,^5^ and comparisons of treatment techniques.^6^

As a tool for assessing the quality of life (QOL) of patients with LL varicose veins, questionnaires can be administered both before and after treatment. There are many different questionnaires for QOL assessment, of which the VEINES-QOL/Sym is one of the most widely used because it has good clinimetric properties and an objective and inexpensive methodology that can be applied in any type of setting and can complement conventional clinical assessment.^7^^-^10

The VEINES-QOL/Sym is a disease-specific self-administered questionnaire with 26 items covering symptoms, performance in activities of daily living, time of day when symptoms are most intense, changes in disease state over the previous year, and psychological impact. The questionnaire produces two scores, one estimating the impact of CVD on QOL, the VEINES-QOL, and another representing the severity of CVD symptoms, the VEINES-Sym. The higher the score, the better the patient’s quality of life.^9^^,^^10^

The objective of this study was to evaluate the impact of surgical treatment on the QOL of patients with LL varicose veins, using the scores of the VEINES QOL/Sym questionnaire, administered before and after surgery.

## METHODS

A before and after study was conducted with all patients who underwent surgical treatment for LL varicose veins at the Vascular Surgery Service run by the Instituto de Medicina Integral Professor Fernando Figueira (IMIP), Recife, PE, Brazil, from December 2013 to July 2014 (sampled consecutively). All of the participants signed free and informed consent forms after being provided with information about the study.

The number of patients enrolled on the study (n = 92) was determined using a formula for sample size calculation based on use of a data collection instrument comprised of categorical items, which is the case of the VEINES/QOL-SYM questionnaire.

The Formula 1 used was:

$$\text{n}=\;\frac{\frac{\left(\begin{array}{c} {\text{c}}^{\text{E}}\\ 2 \end{array}\right)-\;\sum_{\text{i}=1}^{\text{k}}\left(\begin{array}{c} {\text{c}}_{\text{i}}^{\text{E}}\\ 2 \end{array}\right)}{\sum_{\text{i}=1}^{\text{k}}{\text{c}}_{\text{i}}^{\text{o}}}}{1+\;\frac{1}{\text{N}}\text{x}\left[\frac{\left(\begin{array}{c} {\text{c}}^{\text{E}}\\ 2 \end{array}\right)-\;\sum_{\text{i}=1}^{\text{k}}\left(\begin{array}{c} {\text{c}}_{\text{i}}^{\text{E}}\\ 2 \end{array}\right)}{\sum_{\text{i}=1}^{\text{k}}{\text{c}}_{\text{i}}^{\text{o}}}-1\right]}$$ (1)

where: $c^{E}$ = effective number of categories on the data collection instrument; $c_{i}^{E}$ = number of categories in the *i*th item; k = number of items on the data collection instrument; ${\text{c}}_{\text{i}}^{\text{O}}$ = total number of categories in the *i*th item; N = size of population.

A total of 92 patients who underwent surgery for LL varicose veins were assessed. Patients were enrolled on the study at the time of indication of surgical treatment by a vascular surgery specialist, after clinical and ultrasonographic examination. The inclusion criteria were patients at C2 to C6, who were symptomatic and had varicose veins observed during physical examination and on Doppler ultrasonography, with or without saphenous reflux. Patients under the age of 18 years or with clinical comorbidities that contraindicated the surgical procedure were excluded.

Patients were examined standing upright by a trained examiner who classified their lower limbs according to the severity of CVD, using the CEAP classification. When the patient had CVD in both lower limbs, the higher CEAP score was used for analysis.

The surgical technique employed was varicectomy, ligature of perforating veins with reflux (using the conventional technique, with direct access after marking the site with ultrasound guidance), and resection of the saphenous arch, with or without saphenectomy. Saphenectomy was indicated in cases of reflux combined with dilatation of the great or small saphenous veins. All procedures were performed by the same team of surgeons.

All patients wore elastic compression stockings (20-30 mmHg), mid-thigh length (7/8) during the postoperative period and were prescribed an anti-inflammatory (nimesulide) for 5 days and analgesics (dipyrone or paracetamol) to be taken only if in pain.

Although the VEINES-QOL/Sym (Venous Insufficiency Epidemiological and Economic Study) questionnaire can be self-administered, in this study it was administered by a duly trained interviewer in the form of an interview, because of the educational profile of the patients treated at this service (a large proportion of illiteracy and low-educational level). Questionnaires were administered before the surgical procedure (at the time of hospital admission) and again 60 days (±7 days) after surgery.

For the statistical analysis, patients were stratified by CEAP classification and also by age group.

Initially, all variables were analyzed descriptively. Quantitative variables were analyzed using ranges, means, standard deviations, and medians. Qualitative variables were expressed as absolute and relative frequencies.

The Kolmogorov-Smirnov test was used to test the normality of data. The Wilcoxon non-parametric test was used to compare data from before and after surgery, because the assumption of normally distributed data was rejected. Comparisons between two groups were made using the Mann-Whitney non-parametric test. The level of significance was set at 5%.

The study was approved by the Research Ethics Commission at Xxxx, under decision number^.^ 3946-14.

## RESULTS

**Figure 1:**
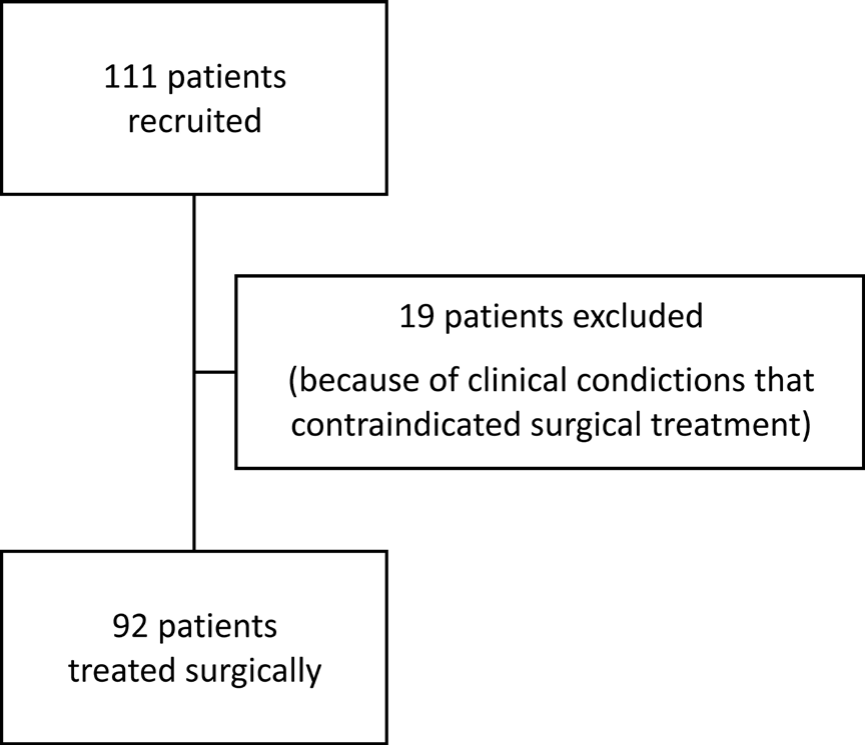
Flow chart of selection of patients for the study.

A total of 118 patients were recruited, 19 of whom were excluded because they had clinical conditions that contraindicated the surgical procedure (Figure 1 – Flow chart). None of the patients operated were lost to follow-up.

A total of 92 patients were assessed, aged from 22 to 71 years (45.71±12.11 years; median: 43.50 years), 76 (82.6%) of whom were female. CEAP clinical class 2 was the most common, in 57.6% (n = 53) of the sample. The clinical and epidemiological data are summarized in Table 1.

**Table 1:** Data on patients and procedures.

**n**	**92**
**Age**	22-71 years
	Mean 45.71±12.11
	Median 43.50
**Sex**	76 F (82.6%)
	34 M (17.4%)
**(C) CEAP Clinical Classification**	2 = 53 (57.6%)
	3 = 20 (21.7%)
	4 = 13 (14.1%)
	5 = 4 (4.3%)
	6 = 2 (2.2%)
**Type of procedure**	V = 63
	V+SAR = 10
	V+S = 15
	V+PL = 4

n = number of patients; V = Varicectomy; V+SAR = Varicectomy + saphenous arch resection; V+S = Varicectomy + Saphenectomy; V+PL = Varicectomy + Perforator ligation.

Patients’ quality of life was assessed using the VEINES-QOL/Sym questionnaire before and after surgery. For both the VEINES-QOL and the VEINES-Sym, higher scores indicate better outcomes.^9^ Table 2 lists descriptive statistics for the scores at the two data collection points. We observed that overall the patients did not exhibit significant differences in QOL between preoperative and postoperative scores, either in terms of improvement of symptoms or of improved QOL.

**Table 2:** Descriptive statistics for preoperative and postoperative VEINES SYM and VEINES QOL scores for the whole sample.

**Variable**	**Assessment**	**n**	**Mean**	**SD**	**Minimum**	**Maximum**	**P25**	**Median**	**P75**	**p** *
SYM	Pre-op.	92	50.00	10.00	29.60	71.25	42.01	49.71	58.47	0.678
	Post-op.	92	50.42	9.92	15.85	61.20	47.44	53.45	57.22	
QOL	Pre-op.	92	50.00	10.00	31.75	72.28	41.58	48.70	56.81	0.809
	Post-op.	92	50.07	10.04	15.57	61.29	46.54	53.37	56.65	

* descriptive level of probability according to Wilcoxon’s nonparametric test.

n = number of patients; SD = standard deviation; P25 = 25^th^ percentile; P75 = 75^th^ percentile.

It was observed that patients with ages in the range of 30 to 40 years did exhibit significant increases in VEINES-SYM and VEINES-QOL scores for the postoperative period, indicating improved symptoms and improved QOL after surgery in this subset of patients. There were no statistically significant changes in the other age groups (Table 3). The Kruskal-Wallis nonparametric test demonstrated that the age groups did not exhibit significant differences at the preoperative (p = 0.269) or postoperative data collections (p = 0.578).

**Table 3:** Descriptive statistics for preoperative and postoperative VEINES SYM and VEINES QOL scores, by age group.

**Age**	**n**	**Variable**	**Assessment**	**Mean**	**SD**	**Minimum**		**Maximum**	**P25**	**Median**		**P75**	**p** *
**< 30**	11	SYM	Pre-op.	48.30	8.37	33.76	62.63		41.82		48.62	53.01	0.534
			Post-op.	50.69	8.67	36.51	59.70		42.19		53.07	58.53	
		QOL	Pre-op.	49.61	8.56	38.41	62.52		44.21		45.29	60.34	0.477
			Post-op.	50.73	6.32	40.95	58.40		44.62		50.25	56.75	
											
**30-40**	20	SYM	Pre-op.	47.53	6.32	37.69	64.36		43.31		46.35	49.58	***0.025***
			Post-op.	51.85	7.06	27.10	59.70		49.39		52.49	56.01	
		QOL	Pre-op.	47.17	6.64	36.05	60.09		41.50		47.28	52.34	***0.048***
			Post-op.	51.96	8.81	18.86	59.90		49.99		54.04	56.73	
											
**40-50**	27	SYM	Pre-op.	52.75	10.99	35.60	73.05		43.51		54.36	60.25	0.597
			Post-op.	50.92	9.74	23.43	59.70		46.42		54.97	58.57	
		QOL	Pre-op.	53.02	11.66	31.95	74.10		41.16		52.69	62.19	0.337
			Post-op.	50.89	9.33	19.07	59.93		48.03		52.25	57.65	
**50-60**	20	SYM	Pre-op.	52.38	12.29	30.22	70.97		42.68		53.45	63.65	0.296
			Post-op.	49.61	10.92	21.63	59.70		46.02		51.26	59.25	
		QOL	Pre-op.	52.24	11.57	34.54	73.26		40.55		52.31	60.17	0.455
			Post-op.	50.14	10.72	20.36	59.93		48.01		52.86	57.00	
											
**>= 60**	14	SYM	Pre-op.	46.16	8.57	33.29	60.23		37.90		47.52	53.36	0.925
			Post-op.	45.58	13.28	13.09	57.91		41.27		51.19	52.77	
		QOL	Pre-op.	45.32	7.00	35.25	55.59		39.22		46.20	52.52	0.975
			Post-op.	44.72	13.25	10.11	56.82		38.26		47.98	56.06	

* descriptive level of probability according to Wilcoxon’s nonparametric test.

n = number of patients; SD = standard deviation; P25 = 25^th^ percentile; P75 = 75^th^ percentile.

There were no statistically significant differences in VEINES-SYM and VEINES-QOL scores from preoperative to postoperative results when patients were divided into groups according to CEAP clinical classification (Table 4). The Kruskal-Wallis nonparametric test demonstrated that the CEAP groups did not exhibit significant differences on VEINES-SYM at the preoperative (p = 0.626) and postoperative (p = 0.400) data collections.

**Table 4:** Descriptive statistics for preoperative and postoperative VEINES SYM and VEINES QOL scores, by severity of clinical status.

**CEAP**	**n**	**Variable**	**Assessment**	**Mean**	**SD**	**Minimum**	**Maximum**	**P25**	**Median**	**P75**	**p** *
**2**	53	SYM	Pre-op.	50.66	9.85	30.22	73.05	43.63	48.53	58.90	0.403
			Post-op.	51.38	8.76	21.63	59.70	48.39	52.94	58.22	
		QOL	Pre-op.	9.98	31.95	73.26	42.26	49.85	57.72	50.43	0.418
			Post-op.	9.14	18.86	59.93	49.42	52.68	56.84	51.14	
**3**	20	SYM	Pre-op.	47.55	10.55	33.29	67.22	38.05	47.39	53.85	0.970
			Post-op.	47.75	12.02	21.55	59.70	37.93	50.80	57.95	
		QOL	Pre-op.	9.44	37.49	67.66	40.38	47.02	58.56	49.06	0.823
			Post-op.	9.81	20.36	59.62	43.31	53.03	57.50	50.10	
**4**	13	SYM	Pre-op.	50.10	8.75	38.07	65.94	43.05	51.13	54.98	0.807
			Post-op.	50.06	6.84	35.06	59.70	46.67	51.88	54.08	
		QOL	Pre-op.	9.74	34.54	68.79	38.52	48.52	54.31	48.06	0.972
			Post-op.	8.99	30.18	58.24	41.39	50.09	56.38	48.43	
**5**	4	SYM	Pre-op.	48.32	15.06	35.16	69.88	36.93	44.12	63.91	0.465
			Post-op.	40.21	19.60	13.09	59.70	20.34	44.03	56.27	
		QOL	Pre-op.	15.98	39.01	74.10	39.80	44.98	67.53	50.77	0.273
			Post-op.	21.05	10.11	58.41	17.37	44.53	56.28	39.39	

* descriptive level of probability according to Wilcoxon’s nonparametric test.

n = number of patients; SD = standard deviation; P25 = 25^th^ percentile; P75 = 75^th^ percentile.

Patients classified as C6 have been excluded from Table 4 because of the small *n* (*n* = 2), which prevented adequate statistical analysis. These patients’ scores did increase during the postoperative period.

None of the subsets exhibited worse QOL postoperatively.

## DISCUSSION

Patients were stratified by the CEAP clinical classification because patients with lower clinical scores theoretically have less venous compromise. The patients were also analyzed by age groups, considering that older patients tend to have more advanced venous disease with a greater impact on quality of life, since CVD is chronic and progressive.

The greater prevalence of LL varicose veins in women observed in this study is consistent with published data. It is important to point out that female sex is one of the risk factors for development of LL CVD.^11^^-^13 The mean age of the patients analyzed was over 40 years. Studies have shown that the prevalence of CVD increases with age, particularly the more severe forms (CEAP 4, 5, and 6).^14^^-^17

Measures of QOL are used as indicators to evaluate the efficacy and impact of specific treatments and also to compare different therapeutic procedures, although other criteria, such as analysis of change in CEAP class, are also used.^18^ In clinical practice, QOL assessment is an important tool, particularly as an outcome variable that can be used to determine the impact that a disease and its treatments have on a person’s life.^14^^,^^15^

Many different questionnaires are available for assessment of QOL in patients with LL varicose veins. The VEINES questionnaire used in this study has good clinimetric properties and objective, inexpensive methodology that can be employed in any setting and complements conventional clinical assessment.^10^^,^^19^^,^^20^ In order to assess QOL, it is necessary to measure reproducible and quantifiable metrics of the disease’s functional, psychological, and social impact. The VEINES-QOL/SYM assesses symptoms, performance of activities of daily living, and the psychological impact of CVD, which is why it was chosen for the present study.

When the VEINES SYM/QOL questionnaire was developed, the CEAP classification was employed for evaluation of the severity of venous disease,^9^ which is the reason for choosing the same classification in this study. The CEAP classification has also been used in other studies of QOL and varicose veins.^5^^,^^6^^,^^21^

When the entire patient sample was analyzed, no significant change in QOL was observed comparing the data collected before and after surgery. A similar result has been described previously by Blomgren et al., in 2006, in a prospective randomized study in which quality of life scores did not exhibit significant improvement over a 2-year observation period after surgery.^5^ This result may be because the majority of the patients in this study were classified as CEAP C2. This raises the hypothesis that the absence of any significant changes in scores after surgery could be because these patients have milder symptoms, having little influence on their QOL.

Several authors have demonstrated the existence of a direct relationship between CVD severity and reduced QOL, predominantly in relation to the physical and functional domains.^9^^,^^22^^-^24 These studies found that people with more severe LL CVD (CEAP 4, 5, and 6) tend to have lower scores on QOL assessment questionnaires.

When the patients were analyzed by age group, the youngest patients (aged 30 to 40 years) exhibited greater postoperative improvement in QOL and a statistically significant increase in QOL was observed in this subset. One hypothesis to explain this result is that this age group has a lower prevalence of related pathologies, which would lead to increased relevance of symptoms secondary to CVD, which, after surgical treatment may undergo significant improvement and, as a consequence, of quality of life. Older people often have other pathological conditions that can also cause LL symptoms and have a negative impact on their QOL.

When patients were analyzed in subsets categorized according to their CEAP clinical classification at preoperative and postoperative assessments, the group of patients with less clinical compromise (C2 and C3) had better QOL after surgery, but the difference did not attain statistical significance. There were no significant differences in QOL before and after surgery in the other subsets.

We found a randomized prospective study^5^ in the literature in which it was observed that even after surgical intervention QOL levels did not improve significantly over a 2-year period, in line with the findings of the present study.

One important issue that should be mentioned and which has been described elsewhere is that it is difficult to study CVD and QOL because of discrepancies between patients’ symptoms, clinical findings, and the results of Doppler ultrasonography. In clinical practice, it is common to see patients with CVD who have the same clinical classification, but distinct physical, functional, and social limitations.^25^^-^30

Quality of life assessment is dependent on each patient’s interpretation of the signs and symptoms of their disease and this is related to their subjective perceptions of their living conditions. The same clinical presentation can cause different functional compromise in different patients or have different emotional and social relevance.^27^ The symptom lower limb pain can be a result of many different diseases and may be erroneously attributed to the presence of varicose veins, as has been described in a prior study.^31^

One of the main limitations of this study lies in the heterogeneous nature of the sample investigated and the treatments provided. Additionally, there were few patients in the more advanced disease classes and, as a consequence, tests to detect statistically significant differences between groups could not be employed. Additionally, no analysis was conducted of correlations between ultrasonographic findings and preoperative and postoperative scores or between CEAP class and patient age. Allocation of patients to subsets may have interfered with analysis of the data because of the low *n* in each group.

## CONCLUSIONS

In the subset of patients aged 30 to 40 years, surgical treatment resulted in improved QOL. In the other subsets, no differences were detected in preoperative and postoperative VEINES-QOL/SYM scores.
