# Supplementary material for: Quality of life assessment before and after surgery for lower limb varicose veins
Source: J Vasc Bras. 2020 May 8;19:e20190108. doi: 10.1590/1677-5449.190108 (PMC8202207; doi:10.1590/1677-5449.190108)
Supplement: Translation

# Translation: Avaliação da qualidade de vida em pacientes portadores de varizes de membros inferiores submetidos a tratamento cirúrgico

**Authors:** Fernanda Appolonio Rocha, Esdras Marques Lins, Catarina Coelho de Almeida, Ronaldo Campelo Dias, Pedro Alberto Livino da Silva, Claudia Almeida Gameleira, Mary Gleyce de Melo Gomes Falcão, José Wellington dos Santos Barros

**Affiliations:** 1 Universidade Federal de Pernambuco – UFPE, Disciplina de Cirurgia Vascular, Recife, PE, Brasil.; 2 Universidade Federal de Pernambuco – UFPE, Hospital das Clínicas, Serviço de Cirurgia Vascular, Recife, PE, Brasil.; 3 Faculdade Pernambucana de Saúde – FPS, Curso de Medicina, Recife, PE, Brasil.; 4 Instituto de Medicina Integral Professor Fernando Figueira – IMIP, Serviço de Cirurgia Vascular, Recife, PE, Brasil.

**Keywords:** qualidade de vida, varizes, insuficiência venosa

## Abstract

**Contexto:**

As varizes dos membros inferiores representam uma das doenças mais prevalentes na população mundial e resultam em grande impacto na qualidade de vida dos pacientes devido às limitações nas atividades diárias e no desempenho funcional. Vários autores têm ressaltado a importância da avaliação da qualidade de vida na abordagem de pacientes portadores de doença venosa crônica.

**Objetivos:**

Avaliar a qualidade de vida antes e após o tratamento cirúrgico de pacientes portadores de varizes dos membros inferiores.

**Métodos:**

Através de estudo de antes e depois, foram avaliados 92 portadores de varizes dos membros inferiores submetidos a tratamento cirúrgico. Os pacientes foram divididos em grupos de acordo com a faixa etária e a classificação clínica CEAP. A qualidade de vida foi avaliada através do questionário VEINES QOL/SYM, aplicado no pré-operatório e 60 dias após a operação.

**Resultados:**

Do total de 92 indivíduos analisados, 82,6% (76) eram mulheres. Com relação à idade, a média foi de 45,7±12,11. A classe CEAP 2 foi a mais encontrada, em 57,6% dos pacientes. Foi encontrada diferença significativa entre os escores antes e após a cirurgia para os pacientes na faixa etária entre 30 e 40 anos. Não houve diferença entre os escores nos momentos pré e pós-operatório entre os grupos CEAP.

**Conclusões:**

Não foi encontrada diferença na qualidade de vida antes e após a cirurgia na maioria dos pacientes do estudo.

## INTRODUÇÃO

A doença venosa crônica (DVC) dos membros inferiores (MMII) é extremamente comum e possui apresentações variáveis. É caracterizada pela disfunção no sistema venoso, secundária à hipertensão venosa, causada por incompetência valvular e/ou obstrução do fluxo venoso. Além de causar comprometimento estético, a DVC pode ocasionar sintomas e levar a complicações e sequelas, que podem influenciar negativamente na qualidade de vida dos seus portadores^1^.

A partir da terceira década de vida, observa-se um aumento importante na incidência de DVC. No Brasil, um estudo epidemiológico realizado por Maffei^2^ encontrou prevalência de 35,5% de varizes e formas graves de DVC. Esse número aumenta com a idade: na faixa etária de 30 a 40 anos, a doença acomete 3% dos homens e 20% das mulheres; aos 70 anos de idade, 70% da população apresenta algum grau de doença venosa^2^^,^^3^.

Embora o tratamento cirúrgico seja um método terapêutico amplamente utilizado para a DVC, há poucos estudos avaliando o impacto na qualidade de vida (QV) dos pacientes submetidos ao mesmo. Existem relatos avaliando a QV após o tratamento cirúrgico, porém relacionando-a a outros fatores, como a hemodinâmica venosa^4^, o uso de ultrassonografia pré-operatória^5^, e comparação de técnicas de tratamento^6^.

Como ferramenta para avaliar a qualidade de vida (QV) de pacientes portadores de varizes nos MMII, questionários podem ser empregados antes e após o tratamento ao qual foram submetidos. Entre os diversos questionários para avaliação de QV, o VEINES-QOL/SYM é um dos mais utilizados por apresentar boas propriedades clinimétricas, além de uma metodologia objetiva e não onerosa que pode ser utilizada em qualquer ambiente e que pode completar a avaliação clínica convencional^7^^-^10.

O VEINES-QOL/SYM consiste em um questionário específico e autoaplicável com 26 itens relacionados aos sintomas, desempenho nas atividades de vida diária, hora do dia que os sintomas são mais intensos, alterações ocorridas em relação à doença no último ano e o seu impacto psicológico. Este questionário produz dois escores, um relacionado à estimativa do impacto da DVC na QV, que é o VEINES- QOL, e outro relacionado aos sintomas decorrentes da DVC, o VEINES-Sym. Quanto maior o escore, melhor é a qualidade de vida do paciente^9^^,^^10^.

A partir destas considerações, o objetivo deste estudo foi avaliar o impacto do tratamento cirúrgico na QV dos pacientes portadores de varizes dos MMII, utilizando os escores do questionário VEINES QOL/Sym, aplicado antes e após a cirurgia.

## MÉTODOS

Foi realizado estudo do tipo antes e depois em todos os pacientes submetidos a tratamento cirúrgico de varizes de membros inferiores no Serviço de Cirurgia Vascular do Instituto de Medicina Integral Professor Fernando Figueira (IMIP), Recife (PE), no período entre dezembro de 2013 e julho de 2014 (amostra consecutiva). Todos os participantes assinaram termo de consentimento livre e esclarecido após receberem informações sobre o estudo.

O número de pacientes incluídos (n = 92) no estudo foi determinado a partir de fórmula para o cálculo amostral baseada na utilização de um instrumento de coleta de dados formado por itens categóricos, como é o caso do questionário aplicado, o VEINES/QOL-SYM.

A Fórmula 1 utilizada foi:

$$\text{n}=\;\frac{\frac{\left(\begin{array}{c} {\text{c}}^{\text{E}}\\ 2 \end{array}\right)-\;\sum_{\text{i}=1}^{\text{k}}\left(\begin{array}{c} {\text{c}}_{\text{i}}^{\text{E}}\\ 2 \end{array}\right)}{\sum_{\text{i}=1}^{\text{k}}{\text{c}}_{\text{i}}^{\text{o}}}}{1+\;\frac{1}{\text{N}}\text{x}\left[\frac{\left(\begin{array}{c} {\text{c}}^{\text{E}}\\ 2 \end{array}\right)-\;\sum_{\text{i}=1}^{\text{k}}\left(\begin{array}{c} {\text{c}}_{\text{i}}^{\text{E}}\\ 2 \end{array}\right)}{\sum_{\text{i}=1}^{\text{k}}{\text{c}}_{\text{i}}^{\text{o}}}-1\right]}$$ (1)

onde: $c^{E}$ = número de categorias efetivas do instrumento de coleta; $c_{i}^{E}$ = número de categorias do i-ésimo item; k = número de itens do instrumento de coleta; ${\text{c}}_{\text{i}}^{\text{O}}$ = número total de categorias do i-ésimo item; N = tamanho da população.

Foram avaliados 92 pacientes submetidos a cirurgia de varizes de MMII. Os pacientes foram incluídos no estudo à indicação de tratamento cirúrgico por médico especialista em Cirurgia Vascular, após avaliação clínica e ultrassonográfica. Os critérios de inclusão foram pacientes de C2 a C6, sintomáticos e com presença de varizes ao exame físico e à ultrassonografia com Doppler, associadas ou não a refluxo de safena(s). Foram excluídos pacientes menores de 18 anos ou com comorbidades clínicas que contraindicassem o procedimento operatório.

Os pacientes foram examinados em posição ortostática por um examinador capacitado que, após a inspeção, classificou os membros inferiores de acordo com a gravidade da DVC, utilizando a classificação clínica CEAP. Quando o paciente era portador de DVC em ambos os membros inferiores, foi considerado o de maior escore do CEAP.

A técnica cirúrgica empregada foi varicectomia, ligadura de perfurantes (técnica convencional, por abordagem direta, após marcação guiada por ultrassonografia) nos casos em que havia refluxo nas mesmas, e crossectomia com ou sem safenectomia. A safenectomia era indicada nos casos de refluxo associado à dilatação da safena magna ou parva. Os procedimentos foram realizados sempre pelo mesmo grupo de cirurgiões.

No período pós-operatório, todos os pacientes usaram meias de compressão elástica (20-30 mmHg), comprimento meia-coxa (7/8) e foram prescritos anti-inflamatório (nimesulida) por 5 dias e analgésico (dipirona ou paracetamol) somente no caso de dor.

O questionário VEINES-QOL/SYM (Venous Insufficiency Epidemiological and Economic Study), apesar de ser autoaplicável, foi aplicado na forma de entrevista, por entrevistador devidamente treinado, ao perfil de escolaridade dos pacientes atendidos no serviço (grande número de analfabetos e de baixa escolaridade). Os questionários foram respondidos previamente ao procedimento cirúrgico (no momento da admissão hospitalar) e após um período de 60 dias (±7 dias) da cirurgia.

Para a análise estatística, os pacientes foram estratificados de acordo com a classificação clínica CEAP e foram ainda divididos em faixas etárias.

Inicialmente todas as variáveis foram analisadas descritivamente. Para as variáveis quantitativas esta análise foi feita observando os valores mínimos e máximos, e por meio do cálculo de médias, desvios padrão e mediana. Para as variáveis qualitativas, foram calculadas as frequências absolutas e relativas.

A normalidade dos dados foi testada através do teste de Kolmogorov-Smirnov. Para a comparação dos momentos pré e pós operatório foi utilizado o teste não paramétrico de Wilcoxon, pois a suposição de normalidade dos dados foi rejeitada. Para a comparação de dois grupos foi utilizado o teste não paramétrico de Mann-Whitney. O nível de significância utilizado para os testes foi de 5%.

O estudo foi aprovado pelo Comitê de Ética em Pesquisa (CEP) do IMIP, sob parecer nº 3946-14.

## RESULTADOS

Forem recrutados 118 pacientes, dos quais 19 foram excluídos por apresentarem condições clínicas que eram contraindicações ao procedimento operatório (Figura 1 – *Flow chart*). Não houve perda de seguimento dos pacientes operados.

**Figura 1:** Flow chart de pacientes da pesquisa.

Foram avaliados 92 pacientes com idade entre 22 e 71 anos (45,71±12,11 anos; mediana de 43,50 anos), dos quais 76 (82,6%) eram do sexo feminino. A classificação clínica CEAP 2 foi a mais presente, em 57,6% (n = 53) da amostra. Os dados clínicos e epidemiológicos encontram-se resumidos na Tabela 1.

**Tabela 1:** Dados dos pacientes e procedimentos realizados.

**n**	**92**
**Idade**	22-71 anos
	Média 45,71±12,11
	Mediana 43,50
**Sexo**	76 F (82,6%)
	34 M (17,4%)
**Classificação Clínica (C) CEAP**	2 = 53 (57,6%)
	3 = 20 (21,7%)
	4 = 13 (14,1%)
	5 = 4 (4,3%)
	6 = 2 (2,2%)
**Tipo de procedimento**	V = 63
	V+C = 10
	V+S = 15
	V+LP = 4

n = número de pacientes; V = Varicetomia; V+C = Varicetomia + crocectomia; V+S = Varicectomia + Safenectomia; V+LP = Varicectomia + Ligadura de perfurantes.

Os pacientes foram avaliados quanto à qualidade de vida por meio do questionário VEINES-QOL/Sym nos momentos pré e pós operatório. Tanto para o VEINES-QOL quanto para o VEINES-Sym, escores maiores indicam melhores desfechos^9^. Na Tabela 2 apresentamos os valores descritivos dos escores nos dois momentos de avaliação. Observamos que os pacientes não apresentam alterações significativas na QV do momento pré para o momento pós-operatório, tanto no que diz respeito à melhora dos sintomas como na melhora da QV.

**Tabela 2:** Valores descritivos (scores) do VEINES SYM e VEINES QOL, nos momentos pré e pós-operatório para o total da amostra estudada.

**Variável**	**Momento**	**n**	**Média**	**DP**	**Mínimo**	**Máximo**	**P25**	**Mediana**	**P75**	**p** *
SYM	Pré	92	50,00	10,00	29,60	71,25	42,01	49,71	58,47	0,678
	Pós	92	50,42	9,92	15,85	61,20	47,44	53,45	57,22	
										
QOL	Pré	92	50,00	10,00	31,75	72,28	41,58	48,70	56,81	0,809
	Pós	92	50,07	10,04	15,57	61,29	46,54	53,37	56,65	

* nível descritivo de probabilidade do teste não paramétrico de Wilcoxon.

n = número de pacientes; DP = desvio padrão, P25 = percentil 25; P75 = percentil 75.

Foi observado que na faixa etária de 30 a 40 anos, quando avaliados pelos questionários VEINES SYM e VEINES QOL, houve um acréscimo significativo do escore no pós-operatório, representando uma melhora nos sintomas e na QV após a cirurgia nesses pacientes. Nas demais faixas não houve alteração estatisticamente significante (Tabela 3). Por meio do teste não paramétrico de Kruskal-Wallis observamos que os grupos etários não apresentaram diferenças significativas nos momentos pré (p = 0,269) e pós-operatório (p = 0,578).

**Tabela 3:** Valores descritivos (escores) do VEINES SYM e VEINES QOL, nos momentos pré e pós-operatório, segundo a faixa etária.

**Idade**	**n**	**Variável**	**Momento**	**Média**	**DP**	**Mínimo**		**Máximo**	**P25**	**Mediana**		**P75**	**p** *
**< 30**	11	SYM	Pré	48,30	8,37	33,76	62,63		41,82		48,62	53,01	0,534
			Pós	50,69	8,67	36,51	59,70		42,19		53,07	58,53	
		QOL	Pré	49,61	8,56	38,41	62,52		44,21		45,29	60,34	0,477
			Pós	50,73	6,32	40,95	58,40		44,62		50,25	56,75	
											
**30-40**	20	SYM	Pré	47,53	6,32	37,69	64,36		43,31		46,35	49,58	***0,025***
			Pós	51,85	7,06	27,10	59,70		49,39		52,49	56,01	
		QOL	Pré	47,17	6,64	36,05	60,09		41,50		47,28	52,34	***0,048***
			Pós	51,96	8,81	18,86	59,90		49,99		54,04	56,73	
											
**40-50**	27	SYM	Pré	52,75	10,99	35,60	73,05		43,51		54,36	60,25	0,597
			Pós	50,92	9,74	23,43	59,70		46,42		54,97	58,57	
		QOL	Pré	53,02	11,66	31,95	74,10		41,16		52,69	62,19	0,337
			Pós	50,89	9,33	19,07	59,93		48,03		52,25	57,65	
**50-60**	20	SYM	Pré	52,38	12,29	30,22	70,97		42,68		53,45	63,65	0,296
			Pós	49,61	10,92	21,63	59,70		46,02		51,26	59,25	
		QOL	Pré	52,24	11,57	34,54	73,26		40,55		52,31	60,17	0,455
			Pós	50,14	10,72	20,36	59,93		48,01		52,86	57,00	
											
**>= 60**	14	SYM	Pré	46,16	8,57	33,29	60,23		37,90		47,52	53,36	0,925
			Pós	45,58	13,28	13,09	57,91		41,27		51,19	52,77	
		QOL	Pré	45,32	7,00	35,25	55,59		39,22		46,20	52,52	0,975
			Pós	44,72	13,25	10,11	56,82		38,26		47,98	56,06	

* nível descritivo de probabilidade do teste não-paramétrico de Wilcoxon.

n = número de pacientes; DP = desvio padrão; P25 = percentil 25; P75 = percentil 75.

Não houve alteração estatisticamente significante dos escores do VEINES-SYM e VEINES-QOL do momento pré para o momento pós-operatório quando os pacientes foram divididos em grupos de acordo com a classificação clínica CEAP (Tabela 4). Por meio do teste não paramétrico de Kruskal-Wallis observamos que os grupos de CEAP não apresentaram diferenças significativas no momento pré (p = 0,626) e no momento pós-operatório (p = 0,406) para o VEINES-SYM; e nos momentos pré (p = 0,843) e pós-operatório (p = 0,400) para o VEINES-QOL.

**Tabela 4:** Valores descritivos (escores) do VEINES SYM e QOL, nos momentos pré e pós operatório, segundo a gravidade do quadro clínico.

**CEAP**	**n**	**Variável**	**Momento**	**Média**	**DP**	**Mínimo**	**Máximo**	**P25**	**Mediana**	**P75**	**p** *
**2**	53	SYM	Pré	50,66	9,85	30,22	73,05	43,63	48,53	58,90	0,403
			Pós	51,38	8,76	21,63	59,70	48,39	52,94	58,22	
		QOL	Pré	9,98	31,95	73,26	42,26	49,85	57,72	50,43	0,418
			Pós	9,14	18,86	59,93	49,42	52,68	56,84	51,14	
**3**	20	SYM	Pré	47,55	10,55	33,29	67,22	38,05	47,39	53,85	0,970
			Pós	47,75	12,02	21,55	59,70	37,93	50,80	57,95	
		QOL	Pré	9,44	37,49	67,66	40,38	47,02	58,56	49,06	0,823
			Pós	9,81	20,36	59,62	43,31	53,03	57,50	50,10	
**4**	13	SYM	Pré	50,10	8,75	38,07	65,94	43,05	51,13	54,98	0,807
			Pós	50,06	6,84	35,06	59,70	46,67	51,88	54,08	
		QOL	Pré	9,74	34,54	68,79	38,52	48,52	54,31	48,06	0,972
			Pós	8,99	30,18	58,24	41,39	50,09	56,38	48,43	
**5**	4	SYM	Pré	48,32	15,06	35,16	69,88	36,93	44,12	63,91	0,465
			Pós	40,21	19,60	13,09	59,70	20,34	44,03	56,27	
		QOL	Pré	15,98	39,01	74,10	39,80	44,98	67,53	50,77	0,273
			Pós	21,05	10,11	58,41	17,37	44,53	56,28	39,39	

* nível descritivo de probabilidade do teste não-paramétrico de Wilcoxon.

n = número de pacientes; DP = desvio padrão; P25 = percentil 25; P75 = percentil 75.

Os pacientes com classificação C6 foram excluídos da Tabela 4 devido ao *n* reduzido (*n* = 2), impossibilitando análise estatística adequada. Nesses pacientes houve aumento dos escores no pós-operatório.

Não foi observada piora da QV em nenhum subgrupo.

## DISCUSSÃO

Os pacientes foram estratificados de acordo com a classificação clínica CEAP, uma vez que pacientes com menor escore clínico, teoricamente, apresentariam menor comprometimento venoso. Os pacientes foram ainda divididos para a análise em faixas etárias, considerando que faixas etárias mais avançadas tendem a possuir doença venosa mais avançada, visto que a DVC é crônica e progressiva, com um impacto maior na qualidade de vida.

A maior prevalência de varizes dos MMII em mulheres encontrada nesta pesquisa está de acordo com os dados da literatura. É importante ressaltar que o sexo feminino é descrito como um dos fatores de risco para o desenvolvimento da DVC dos MMII^11^^-^13. A média de idade dos pacientes avaliados foi superior a 40 anos. Estudos têm evidenciado que a prevalência da DVC aumenta com a idade, sobretudo em suas formas mais graves (CEAP 4, 5 e 6)^14^^-^17.

As informações sobre QV têm sido utilizadas tanto como indicadores para avaliação da eficácia e impacto de determinados tratamentos quanto na comparação entre diferentes procedimentos terapêuticos, embora outros critérios, como a avaliação da mudança de classe CEAP também sejam empregados^18^. A avaliação da QV na prática clínica é uma ferramenta importante, particularmente como uma variável de desfecho capaz de verificar o impacto da doença e de seu tratamento na vida do indivíduo^14^^,^^15^.

Na avaliação da QV em pacientes portadores de varizes dos MMII, encontram-se disponíveis diversos questionários. O questionário VEINES, utilizado nesta pesquisa, apresenta boas propriedades clinimétricas, além de uma metodologia objetiva, não onerosa, praticável em qualquer recinto, e que completa a avaliação clínica convencional^10^^,^^19^^,^^20^. Para avaliar a QV é necessário aferir medidas quantificáveis reprodutíveis do impacto funcional, psicológico e social da doença avaliada. O VEINES-QOL/SYM avalia os sintomas, o desempenho nas atividades de vida diária e o impacto psicológico da DVC, o que justificou a escolha da utilização desse questionário no presente estudo.

O questionário VEINES SYM/QOL, quando desenvolvido, utilizou a classificação CEAP para a avaliação da gravidade da doença venosa^9^; motivo pelo qual foi utilizada a mesma classificação neste estudo. A classificação CEAP foi também utilizada em outros estudos sobre QV e varizes^5^^,^^6^^,^^21^.

Quando considerados todos os pacientes, não foi observada alteração significativa da QV antes e depois do procedimento cirúrgico. Resultado semelhante foi descrito previamente por Blomgren et al. em 2006, quando apresentaram um estudo prospectivo randomizado no qual os escores de qualidade de vida após a cirurgia não apresentaram melhora significativa durante um período de observação de dois anos^5^. Este resultado pode ser explicado porque a maior parte dos pacientes deste estudo foi classificada, segundo a classificação clínica CEAP, como C2. Pode ser levantada a hipótese de que a ausência de alteração significativa dos escores após a cirurgia deve-se ao fato desses pacientes apresentarem sintomas mais leves, levando a uma menor influência na QV desses indivíduos.

Vários autores demonstraram a existência de uma relação direta entre a gravidade da DVC e a diminuição da QV, sobretudo em relação aos aspectos físicos e funcionais^9^^,^^22^^-^24. Esses estudos apontaram que indivíduos com maior gravidade da DVC dos MMII (CEAP 4, 5 e 6) tendem a apresentar escores mais baixos nos questionários de avaliação da QV.

Quando os pacientes foram avaliados segundo a faixa etária, os mais jovens (entre 30 e 40 anos) apresentaram a maior melhora da QV no pós-operatório, sendo observado um aumento da QV estatisticamente significativo nesse grupo. Uma das hipóteses para justificar tal resultado seria a menor prevalência de patologias associadas nessa faixa etária, o que levaria a uma maior relevância dos sintomas secundários à DVC nesses pacientes, e que com o tratamento cirúrgico puderam ter uma melhora significativa desses sintomas e, consequentemente, da qualidade de vida. Indivíduos com mais idade frequentemente apresentam outras condições patológicas que podem resultar em sintomas nos MMII, influenciando assim negativamente na QV.

Quando os pacientes foram avaliados em grupos de acordo com a classificação clínica do CEAP nos momentos pré e pós-operatório, o grupo de pacientes de menor comprometimento clínico (C2 e C3) apresentou melhor QV após a cirurgia, porém sem significância estatística. Não houve diferença significativa entre QV dos demais grupos, antes e depois da cirurgia.

Encontramos na literatura um estudo prospectivo randomizado^5^ no qual foi observado que mesmo após intervenção cirúrgica, os níveis de QV não melhoraram significativamente durante um período de 2 anos, assim como os achados da presente pesquisa.

Um aspecto importante a ser ressaltado e que tem sido descrito, é a dificuldade em estudar DVC e QV devido à discrepância entre os sintomas do paciente, os achados clínicos, e os resultados da ultrassonografia com Doppler. Na prática clínica é comum encontrarmos pacientes com DVC de mesma classificação clínica, mas que apresentam limitações físicas, funcionais e sociais distintas^25^^-^30.

A avaliação da QV depende da interpretação que cada paciente faz dos sinais e sintomas da sua doença e está relacionada à percepção subjetiva de suas condições de vida. O mesmo quadro clínico para indivíduos diferentes pode resultar em perdas funcionais distintas, ou ter relevâncias emocionais e sociais diversas^27^. O sintoma dor nos membros inferiores pode ser decorrente de diversas outras doenças e pode estar relacionado à presença de varizes de forma equivocada, conforme já descrito em estudo prévio^31^.

Uma das principais limitações do estudo encontra-se na heterogeneidade do grupo avaliado, assim como dos tratamentos realizados. Além disso, no presente estudo o número de pacientes de classes mais avançadas foi reduzido e, por consequência, a detecção de diferença estatística significativa entre os grupos não pôde ser realizada. Além disso, não houve avaliação da correlação dos achados ultrassonográficos com os escores pré e pós-operatórios e não foi realizada a análise da correlação da classe CEAP e a idade dos pacientes. A divisão dos pacientes em subgrupos pode ter dificultado a análise dos dados devido ao *n* reduzido em cada grupo.

## CONCLUSÃO

No grupo de pacientes entre 30 e 40 anos, o tratamento cirúrgico proporcionou uma melhora na QV. Nos demais grupos, não foi encontrada diferença nos escores do VEINES-QOL/SYM antes e após a cirurgia.
